# Concurrent and Predictive Validity of an Exercise-Specific Scale for the Perception of Velocity in the Back Squat

**DOI:** 10.3390/ijerph191811440

**Published:** 2022-09-11

**Authors:** Ruggero Romagnoli, Sergio Civitella, Carlo Minganti, Maria Francesca Piacentini

**Affiliations:** 1 Department of Human Movement and Health Sciences, University of Rome “Foro Italico”, 00135 Rome, Italy; r.romagnoli2@studenti.uniroma4.it (R.R.); s.civitella@studenti.uniroma4.it (S.C.); carlo.minganti@uniroma4.it (C.M.); 2 Italian Weightlifting Federation ‘FIPE’, 00135 Rome, Italy

**Keywords:** resistance training, velocity-based training, autoregulation training, 1-RM, load-velocity, concurrent validity, reliability, perceived velocity scale

## Abstract

Background: the aim of the study was to develop and validate a specific perception velocity scale for the Back Squat exercise to discriminate the velocity of each repetition during a set. Methods: 31 resistance trained participants completed 3 evaluation sessions, consisting of 3 blinded loads (light, medium, heavy). For each repetition, barbell mean velocity (Vr) was measured with a linear position transducer while perceived velocity (Vp) was reported using the Squat Perception of Velocity (PV) Scale. Results: Pearson correlation coefficients (r) showed very high values for each intensity in the 3 different days (range r = 0.73–0.83) and practically perfect correlation for all loads (range r = 0.97–0.98). The simple linear regression analysis between Vp and Vr revealed values ranging from R^2^ = 0.53 to R^2^ = 0.69 in the 3 intensities and values ranging from R^2^ = 0.95 to R^2^ = 0.97 considering all loads. The reliability (ICC_2__.1_, SEM) of Vp was tested for light (0.85, 0.03), medium (0.90, 0.03) and heavy loads (0.86, 0.03) and for all loads (0.99, 0.11). The delta score (ds = Vp − Vr) showed higher accuracy of the PV at heavy loads. Conclusions: these results show that the PV Squat Scale is a valid and reliable tool that can be used to accurately quantify exercise intensity.

## 1. Introduction

International guidelines on physical activity recommend regular participation in resistance training as it is essential for health [1]. The multiple benefits range from musculoskeletal, cardiometabolic and mental health [2]. Overall, this type of activity brings improvements in quality of life, general health status and is also associated with a decrease in mortality rates [3,4]. In order to achieve the specific predetermined adaptations it is essential to correctly prescribe the training parameters such as intensity and volume [5].

Velocity-based training (VBT) is an objective method to manage and quantify resistance training. In the last few years, VBT is increasingly used as a method to optimize resistance training and avoid excessive fatigue in athletes [6] and utilized in different sport disciplines. Through the relationship between the load lifted and movement velocity, it is possible to create load-velocity profiles that allow the estimation of the 1-repetition maximum (1RM) in resistance training exercises [6,7]. The possibility to visualize barbell velocity during the execution of the exercises, allows coaches to objectively monitor and adjust in real time the intensity (% 1RM) (through the velocity targets) and volume (through the loss of velocity within a set or between multiple sets) of resistance training sessions [8]. This method has been shown to carry out individualized and qualitative training sessions by optimizing time and directing the training towards the desired adaptations [9]. Moreover, the velocity loss observed across repetitions provides an indication of neuromuscular fatigue [10,11] and allows a better management of overall fatigue in athletes. The great popularity of VBT is due to the fact that this method uses real-time velocity data to monitor and possibly adjust the load prescription [12,13]. Although a wide range of different technologies have been used to develop smaller and cheaper devices, being anchored to these devices represents a problem and a limitation especially in the case of team sports or in any case of multiple athletes training at the same time.

To overcome this problem, recently Bautista and colleagues developed a “Perception of Velocity” scale (PV scale) and demonstrated, first in the bench press [14] and then in the back squat [15], that after a short period of familiarization with the combined use of the scale (subjective parameter) and an electronic device (objective parameter), participants were able to estimate the average movement velocity of a set even without objective feedback. It was later shown that this subjective parameter still shows room for improvement. Romagnoli et al. [16] in fact reported that 5 weeks of specific training utilizing an electronic device and the PV scale increased the accuracy of the perception of barbell velocity compared to baseline values.

Moreover, the range of velocities within a scale should be exercise specific. In fact, the velocity associated with the 1RM load, called the minimum velocity threshold (MVT), is exercise specific and has been shown to differ between exercises [17]. For example in the Prone Bench Pull the MVT is about 0.50 m·s^−1^ [18], in the Squat about 0.30 m·s^−1^ [19] while in the Bench Press about 0.15 m·s^−1^ [20]. For this reason, displaying 0.1 as the lowest value while performing a squat exercise could lead to an underestimation of the velocity at both submaximal and maximal loads. In addition, in the squat load-velocity profile, a variation of 0.1 m·s^−1^ corresponds to a load variation of approximately 10% of 1RM or more [21]. Therefore, displaying a scale with only 0.1 m·s^−1^ intervals can be misleading in the correct velocity estimation. While the existing PV scale [14] has the purpose to perceive the average velocity of a whole set, it can be hypothesized that, in line with the principles of VBT, more intermediate values can help athletes discriminate between one repetition and the next within a same set.

Therefore, the aim of the present study was to develop and validate a specific perception velocity scale for the Back Squat exercise to be used to discriminate the velocity of each repetition during a set. This scale can be useful for athletes and non-athletes, for a correct approach to resistance training, in order to develop performance, physical fitness and health and avoid any maladaptation.

## 2. Materials and Methods

### 2.1. Study Design

This research was designed to investigate the validity and reliability of a specific perception velocity scale designed for the Squat exercise. All subjects undertook two familiarization sessions and one 1RM test, followed by three experimental trials. The sessions were held on days separated by at least 48 h. A Certified Strength and Conditioning Specialist (CSCS— NSCA) and 3 spotters ensured safety and proper technical execution during each training session. Prior to each session, bodyweight exercises, mobility, and dynamic stretching was then followed by a specific warm-up consisting of sets with progressive loading. Participants received strong verbal encouragement during test sessions.

### 2.2. Subjects

In this case, 31 resistance-trained volunteers (17 females, 14 males) participated in this investigation. Subjects’ characteristics are presented in Table 1. Recruited subjects had at least 2 years of resistance training experience and a minimum squat frequency of once per week. Inclusion criteria were: (i) no previous experience in VBT; (ii) no muscle or bone injury before or during the intervention period. All participants received detailed information regarding the procedures and signed a written informed consent. The study protocol adhered to the Declaration of Helsinki and was approved by the Institutional Review Board (CAR. 75/2021).

The sample size was estimated through an a-priori power analysis carried out with the G*Power software (G*Power V 3.1.9.7 Franz Faul, Universität Kiel, Kiel, Germany). For the procedure the following parameters were taken into account α = 0.05 and a power = 0.80.

### 2.3. Procedures

#### 2.3.1. Squat PV Scale

**Figure 1:**
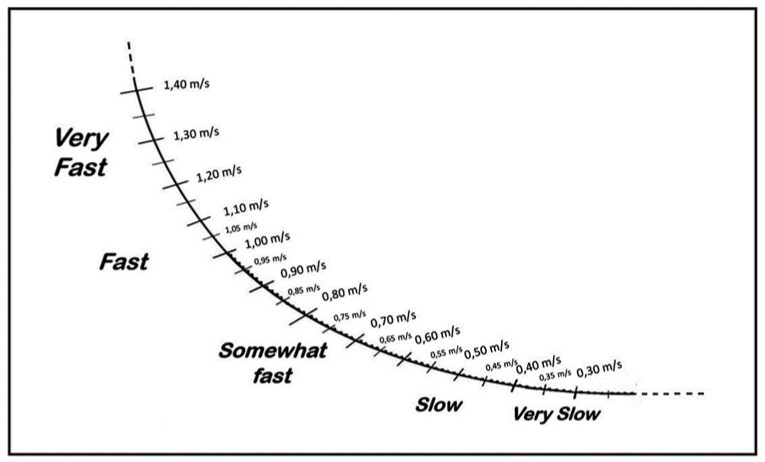
Perception Velocity Scale for Squat.

The scale (Figure 1) has a range of numerical values from 0.30 to 1.4 m·s^−1^ that are consistent with what reported in the literature as the minimum and maximal velocity threshold for the squat exercise [21,22]. Interval values are close to the second decimal point. Moreover, a second order polynomial regression model was used.

The verbal anchors are all associated with how an athlete would perceive the movement of the barbell from very fast to very slow.

#### 2.3.2. Familiarization Sessions

In addition to recording the descriptive characteristics of the participants and standardizing the lifting technique, the first two sessions were used to familiarize with the specific squat perception of velocity scale during a training session.

Mean propulsive velocity of each repetition, which from now on we will consider as “real velocity” (Vr) was recorded through a linear position transducer (LPT) (Vitruve, SPEED4LIFTS S.L., Madrid, Spain).

On the first day, medium-high loads were prescribed to familiarize participants with the lower part of the scale, (i.e., relating to medium-low velocities). During the second day medium-high velocities were prescribed through the use of lighter loads, in order to familiarize participants with the medium-high part of the scale.

In this phase, participants received visual and auditory feedback from the LPT (Smartphone App) in real time as they performed the repetitions. At the end of the set, the maximum and minimum velocity values achieved were provided and subjects were asked to visualize them on the scale for anchoring.

#### 2.3.3. One Repetition Maximum

Each participant underwent a maximal incremental test for squat exercise performed with free weights, 48–72 h after the familiarization sessions. The required execution and test protocol are the same used in a previous investigation [16]. Briefly, the increases in load were assessed based on the mean velocity of the barbell. 1RM was determined as the highest load the subject was able to lift once with a correct technique.

This session can also be considered as a familiarization session because the subjects received feedback of their Vr from the LPT and, at the end of each set, they visualized the velocities on the scale.

#### 2.3.4. Perception Velocity Evaluation Sessions

The accuracy in perceiving velocity (Vp) was assessed during a test with 3 blinded loads (heavy: Vr ≤ 0.4 m·s^−1^, medium: 0.6–0.8 m·s^−1^, light: ≥ 1 m·s^−1^) (Table 2) in a random order on 3 different days. The loading of the barbell was carried out while the subject was outside the weight room and the discs were covered with black sheets in order to blind the subject on the load that was chosen. Thereafter, the subject returned to the weight room without looking at the barbell and performed 2 repetitions at maximum velocity for each load without receiving any kind of velocity feedback from the LPT. At the end of the set, subjects were required to report the perceived velocity (Vp) for each repetition from the PV scale.

### 2.4. Statistical Analysis

Data are presented as mean ± standard deviation. The normal distribution of the data was verified by the Shapiro-Wilk test. Concurrent validity was examined through the Pearson correlation coefficients (r) with 95% confidence limits between Vp and Vr in the 3 testing sessions at 3 different intensities (light, medium and heavy loads). Pearson r magnitudes were interpreted as: trivial (<0.1); small (0.1–0.3); moderate (0.3–0.5); high (0.5–0.7); very high (0.7–0.9); practically perfect (>0.9) [23]. Predictive validity was determined by a simple linear regression between Vp (predictor variable) and Vr (criterion variable). The reliability of the Vp was assessed for each individual load using intraclass correlation coefficient (ICC: model 2, form 1) and Standard Error of Measurement (SEM), which was calculated as follows: SEM = SD × √(1-ICC). The agreement between Vr and Vp was explored using the Bland-Altman plots. The difference between the perceived velocity (Vp) and the real velocity (Vr) was calculated through the delta score (ds): ds = Vp − Vr. Delta score was examined with two factors (Day × Intensity) using a repeated measures ANOVA. Significant main effects were subsequently analyzed using a Bonferroni post hoc test. Effect size were calculated as partial eta-squared (η^2^_p_) and values are interpreted as follows: large (0.14), medium (0.06) and small (0.01) effects [24]. The alpha level was set at *p* < 0.05. Statistical analyses were performed in Microsoft Office Excel® (Microsoft Inc., Redmond, WA, USA) and SPSS v25 (SPSS Inc., Chicago, IL, USA).

## 3. Results

The subjects recruited for the present study, 14 males and 17 females, were experienced in resistance training with a strength level (1-RM/BW ratio) greater than 1.5, and their characteristics are shown in Table 1. Table 3 displays the mean values (± standard deviation) of Vr and Vp recorded over the 3 days for each of the 3 intensities considered.

Pearson correlation coefficients (r) and the coefficients of determination (R^2^) deriving from the correlation analysis and the simple linear regression analysis, respectively, are shown in Table 4 both for each intensity and in all loads in the 3 days of testing. The results show excellent correlation values ranging from 0.73 to 0.83 considering the 3 intensities and between 0.97 and 0.98 considering all loads (Table 4).

The reliability values (ICC_2.1_, SEM) are reported in Table 5 and show similar values between the intensities considered. Considering all the loads, the ICC_2.1_ value obtained was 0.99.

Bland-Altman plots (Supplementary Materials Figure S1) were used to compute the systematic bias and the limits of agreement (ranged between bias − 1.96 × SD and bias + 1.96 × SD) for each of the three loads in the 3 days. The values obtained in light loads were: Day 1 = 0.068 (−0.041, 0.177) m·s^−1^, Day 2 = 0.069 (−0.039, 0.177) m·s^−1^, Day 3 = 0.068 (−0.019, 0.155) m·s^−1^; in medium loads: Day 1 = 0.058 (−0.041, 0.179) m·s^−1^, Day 2 = 0.072 (−0.027, 0.171) m·s^−1^, Day 3 = 0.055 (−0.060, 0.170) m·s^−1^ and in heavy loads: Day 1 = 0.020 (−0.080, 0.120) m·s^−1^, Day 2 = 0.008 (−0.105, 0.120) m·s^−1^, 0.008 (−0.084, 0.100) m·s^−1^.

**Figure 2:**
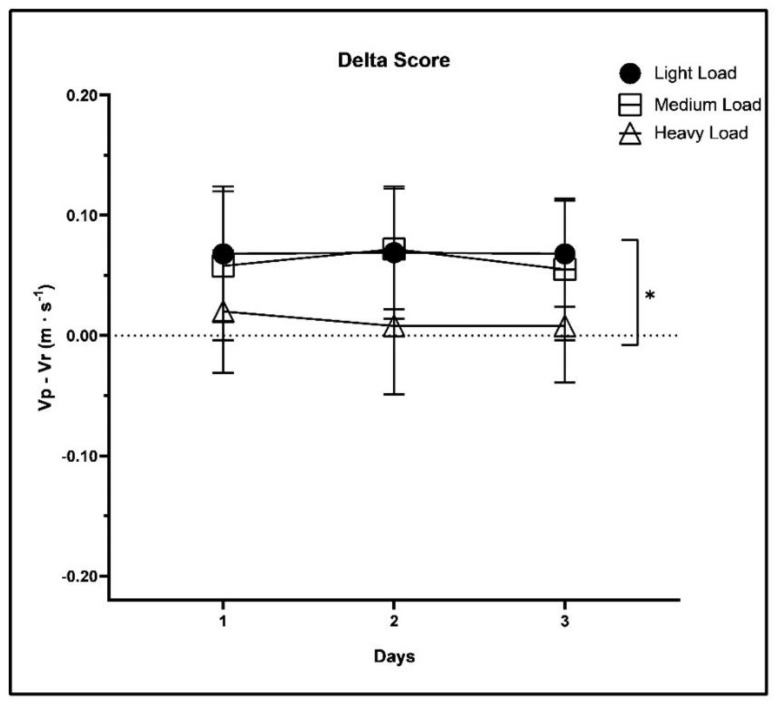
Mean values of the delta score (ds) for the three intensities considered in the three testing sessions. * Significant differences between light and heavy load (*p* < 0.001) and between medium and heavy load (*p* < 0.001).

The delta score (ds), calculated as Vp − Vr, is an easy to interpret parameter to understand the accuracy of the PV. A positive value indicates an overestimation while a negative value indicates an underestimation of the real velocity. The closer this parameter is to zero, the higher the precision of individual perception [16]. Figure 2 shows the trend of the delta score for each of the 3 intensities in the 3 days analyzed. The values (mean ± standard deviation) of the ds were: 0.07 ± 0.06 (light loads, day 1), 0.07 ± 0.05 (light loads, day 2), 0.07 ± 0.04 (light loads, day 3), 0.06 ± 0.06 (medium loads, day 1), 0.07 ± 0.05 (medium loads, day 2), 0.06 ± 0.06 (medium loads, day 3), 0.02 ± 0.05 (heavy loads, day 1), 0.01 ± 0.06 (heavy loads, day 2), 0.01 ± 0.05 (heavy loads, day 3).

The repeated measures of ANOVA showed significant differences in the Intensity factor (*p* < 0.001; η^2^_p_ = 0.55), while for the Day factor (*p* = 0.304; η^2^_p_ = 0.04) and for the Day × Intensity interaction (*p* = 0.108; η^2^_p_ = 0.12) no significant differences were found. Post hoc comparisons revealed a significant difference in ds between light and heavy loads (*p* < 0.001) and between medium and heavy loads (*p* < 0.001), not between light and medium loads (*p* = 0.978).

## 4. Discussion

The aim of the present study was to develop and validate a specific PV Scale to be used for the squat exercise only. To validate this scale, we tested thirty-one resistance trained subjects on three different days for each exercise load range using the Vr, measured with an LPT, as a criterion variable and found very high correlations in the individual intensities tested (Table 4). The results showed a good reliability (0.85–0.90) when the 3 intensities were analyzed separately and an excellent reliability (0.99) considering all the loads (Table 5) [25]. Considering the agreement between Vp and Vr of the 3 loads, very similar values were found in the 3 days of evaluation (Supplementary Materials Figure S1). The data that most of all gives us immediate information on the accuracy of perceived velocity is the delta score, which represents the difference between the value of Vp and that of Vr. The lower the ds the more accurate the perceived velocity. The values obtained are graphically reported in Figure 2, which clearly show that in line with previous studies [16] the lower ds is reported at heavy loads while the light and medium loads had similar values over the 3 days. No significant differences in *Day x Intensity* interaction were seen, showing how the ds does not change in the different experimental sessions.

Recently, in two different studies [14,15] the concurrent validity of a velocity perception scale has been used to measure exercise intensity, for the bench press and the squat. In both cases the participants, carried out a familiarization period in which both objective (device, LPT) and subjective (PV scale) methods were used, and different intensities tested. Subjects were then asked to report the mean velocity value of the set through the PV scale, without knowing the value recorded by the device. The authors reported that after only 1 familiarization session, a high correlation was found between Vr and Vp. However, a following study demonstrated that, the accuracy of the perception of velocity can be further improved [16]. In fact, the accuracy of the perceived velocity was tested (on squat and bench press in the same session) after only 1 day of familiarization and after 5 weeks (2 sessions per week) of combined training with PV scales and linear encoders. The control group instead, that trained without velocity feedback, showed a slightly worse accuracy in the perception of barbell velocity. The existing scale has been used and validated to instruct athletes to perceive the velocity of a whole set and not of every single repetition during the set. The purpose of VBT is to creates individual profiles, autoregulation of intensity and volume and fatigue management [6,7]. In addition, it provides visual feedback that increases motivation and competitiveness and has positive effects on resistance training performance [26,27,28,29]. Since loss of velocity correlates with fatigue during resistance training [6,30], it is important that athletes correctly perceive each single repetition.

Moreover, some athletes that use the maximum and minimal values as reference points to perceive their own velocity might be disturbed by a fixed range of velocities. Creating an exercise-specific PV scale could facilitate learning and improve accuracy in estimation velocity and thus reduce the time to use the combined methods [16,31]. In this regard, we have made changes to the scale to make it specific for squat exercise.

### 4.1. Numerical Values (Max and Min Velocity Threshold)

The scale that was originally developed for the bench press ranged from 0.1 to 1.6 m·s^−1^. However, it has to be pointed out that the same velocity ranges cannot be utilized for all exercises, because the load-velocity profiles are exercise dependent [32,33]. In fact, 1.6 m·s^−1^ represents an extremely high mean velocity value which is not reached in all exercises. This value, in an estimated load-velocity profile, represents about 10–20% 1RM in the bench press exercise [34]. A percentage of load so low that it is practically never used in resistance training. Furthermore, considering that the standard weight of an empty barbell is 20 kg for males and 15 for females, it would already represent 10% of a 200 kg 1RM and 20% of a 100 kg 1RM in the bench press exercise.

Another important parameter which is exercise dependent is the velocity associated with the maximal load (1RM), i.e. the minimum velocity threshold (MVT) [17]. The MVT in the back squat is approximately 0.30 m·s^−1^ [19], therefore displaying the minimum value of 0.10 on the scale could lead participants to underestimate submaximal and maximal loads. For this reason, we have used 0.30 as the MVT.

Moreover, we included interval values close to the second decimal (0.00). In our opinion this is a crucial point for an accurate estimation of barbell velocity. First of all because the values shown by the devices are always approximated to the second decimal. Secondly, a variation of the mean velocity of 0.10 m·s^−1^ corresponds, in the squat, to a variation of load of about 10% 1RM [21,22]. Therefore, a scale with intervals of 0.1 (0.1, 0.2, 0.3, etc.) could actually be misleading in the correct perception of the load to be utilized. Including intervals of 0.01 up to the value of 1.00 m·s^−1^, seems to be more appropriate and accurate in selecting the correct load.

### 4.2. Polynomial Regression Model

Although several studies have investigated load-velocity profiles using both linear [7,18] and second-order polynomial [26,27] regression models, validating both [28,29], we decided to use the latter as it is better represents real world situations. In fact, both in training and during 1-RM tests, after using very light loads, substantial weight increases are made to carry out the following sets with a significant variation in velocity. With heavy loads, also if moved at the maximum possible velocity, there will be small variations registered within a set. With light loads instead, the range of velocities within a set may be very high and this seems to be better captured by this regression model.

### 4.3. Verbal Anchors

We modified the verbal anchors so that they would refer only to velocity, substituting the existing “power zone” with “Somewhat Fast”. When verbal descriptors are used, the terminology used is very important, as these serve to better understand the purpose of the scale and the different terminology used can influence the interpretation of the scale [35,36]. It is also important to have coherent verbal anchors positioned correctly according to their quantitative meaning [37]. For this reason, all verbal anchors refer to a perceived velocity and not to a potential training zone.

To summarize, a scale that detects more precisely each single repetition (and not the set) could be important for a correct interpretation of the velocity-based training where the velocity loss from one repetition to the other could be improved to facilitate its use and consequently reduce the familiarization period necessary for learning.

The main limitations of the study are related to the sample considered. The results obtained are valid for a trained and experienced population in the squat exercise, however we do not know the PV level of novice or untrained subjects. In addition, the PV was evaluated in an isolated condition, after a standardized warm-up. However, we do not know if this parameter can undergo alterations due to physical or mental fatigue conditions generated for example by a previous workout. The strengths of the study are to have added new knowledge in the field of perception of barbell velocity and to have provided a useful practical tool to support existing technology.

## 5. Conclusions

The main purpose of the present study was to develop and validate a specific perception velocity scale for the Back Squat exercise to be used to discriminate the velocity of each repetition during a set. The Perceived Velocity (Vp) reported by the subjects for each repetition performed, as well as being highly correlated with the real velocity (Vr), measured by LPT, showed good reliability for light, medium and heavy loads. It also showed a good degree of accuracy, especially in heavy loads.

These results show that, despite the short familiarization period, the PV Squat Scale is a valid and reliable tool that can be used to accurately quantify exercise intensity. In this way it is possible to carry out individualized and qualitative training sessions by optimizing time and directing the training towards the desired adaptations. The correct use of this scale represents an alternative and inexpensive method of monitoring and individualizing training in order to promote and develop performance, physical fitness and health by avoiding overtraining or incorrect prescription of training parameters.

## Figures and Tables

**Table 1:** Anthropometric characteristics and strength levels (mean ± SD) of the participants (n = 31).

	Age (Years)	Body Mass (kg)	Height (cm)	1-RM (kg)	1-RM/BW
Men (n = 14)	28.7 ± 7.5	80.5 ± 14.4	178 ± 4.6	136.9 ± 26.3	1.71 ± 0.27
Women (n = 17)	23 ± 2.4	56.9 ± 5.4	163.9 ± 4.6	91.1 ± 13	1.63 ± 0.31

**Table 2:** Blinded load test during the Perception of Velocity testing sessions.

Blinded Load Test	
Protocol	
Load	MPV (m·s^−1^)
Light	≥1
Medium	0.6–0.8
Heavy	≤0.4

**Table 3:** Perceived velocity (Vp) and real velocity (Vr), in m·s^−1^, for the three intensities considered in the three testing sessions.

Day	Light Load				Medium Load				Heavy Load			
	Vp		Vr		Vp		Vr		Vp		Vr	
	Mean (SD)	CI (95%)	Mean (SD)	CI (95%)	Mean (SD)	CI (95%)	Mean (SD)	CI (95%)	Mean (SD)	CI (95%)	Mean (SD)	CI (95%)
1	1.05 (0.09)	1.03 1.08	0.99 (0.08)	0.97 1.00	0.65 (0.10)	0.62 0.68	0.59 (0.07)	0.58 0.61	0.39 (0.09)	0.37 0.41	0.37 (0.07)	0.35 0.39
2	1.05 (0.07)	1.03 1.07	0.98 (0.08)	0.96 1.00	0.66 (0.08)	0.64 0.69	0.59 (0.07)	0.57 0.61	0.38 (0.09)	0.36 0.40	0.37 (0.07)	0.35 0.39
3	1.05 (0.07)	1.03 1.07	0.98 (0.07)	0.97 1.00	0.64 (0.09)	0.62 0.66	0.58 (0.07)	0.57 0.60	0.36 (0.08)	0.34 0.38	0.36 (0.08)	0.34 0.37

Values are shown as means (±standard deviation). CI 95% = mean confidence interval (lower–upper limit).

**Table 4:** Pearson correlation coefficients (r) and coefficient of determination (R^2^) between mean perceived velocity (Vp) vs. mean real velocity (Vr).

Day	Light Load		Medium Load		Heavy Load		All Loads	
	r	R^2^	r	R^2^	r	R^2^	r	R^2^
1	0.80 *	0.64	0.81 *	0.65	0.83 *	0.69	0.98	0.96
2	0.73 *	0.53	0.80 *	0.65	0.79 *	0.63	0.97	0.95
3	0.82 *	0.68	0.74 *	0.54	0.82 *	0.68	0.98	0.97

* *p* < 0.01.

**Table 5:** Intraclass Correlation Coefficient (ICC: model 2, form 1) and Standard Error of Measurement (SEM).

Loads	ICC (95%IC)	SEM
Light	0.85 (0.77–0.90)	0.03
Medium	0.90 (0.85–0.94)	0.03
Heavy	0.86 (0.79–0.91)	0.03
All	0.99 (0.99–0.99)	0.11

## Data Availability

Data available on request due to restrictions (privacy).

## Supplementary Materials

The following supporting information can be downloaded at: https://www.mdpi.com/1660-4601/19/18/11440/s1, Figure S1: Bland-Altman Plots. Agreement between Vp and Vr for light, medium and heavy loads in the 3 days of evaluation.
