# Supplementary material for: Concurrent and Predictive Validity of an Exercise-Specific Scale for the Perception of Velocity in the Back Squat
Source: Int J Environ Res Public Health. 2022 Sep 11;19(18):11440. doi: 10.3390/ijerph191811440 (PMC9517416; doi:10.3390/ijerph191811440)
Supplement: Supplementary file 1 [file ijerph-19-11440-s001.zip › ijerph-1885601-supplementary.pdf]

Supplementary materials – S1: Bland-Altman Plots. Agreement between Vp and Vr for light, medium and heavy loads in the 3 days of evaluation.

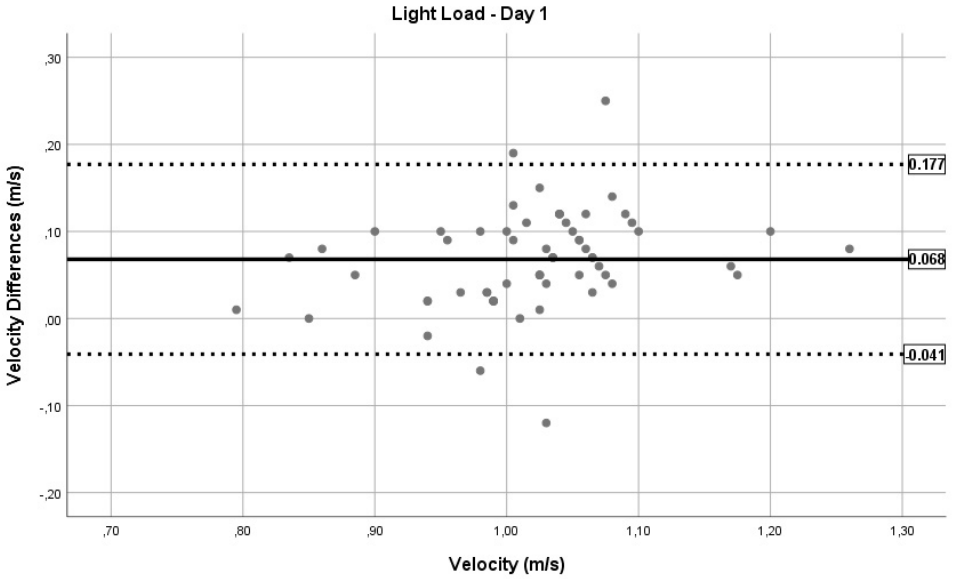

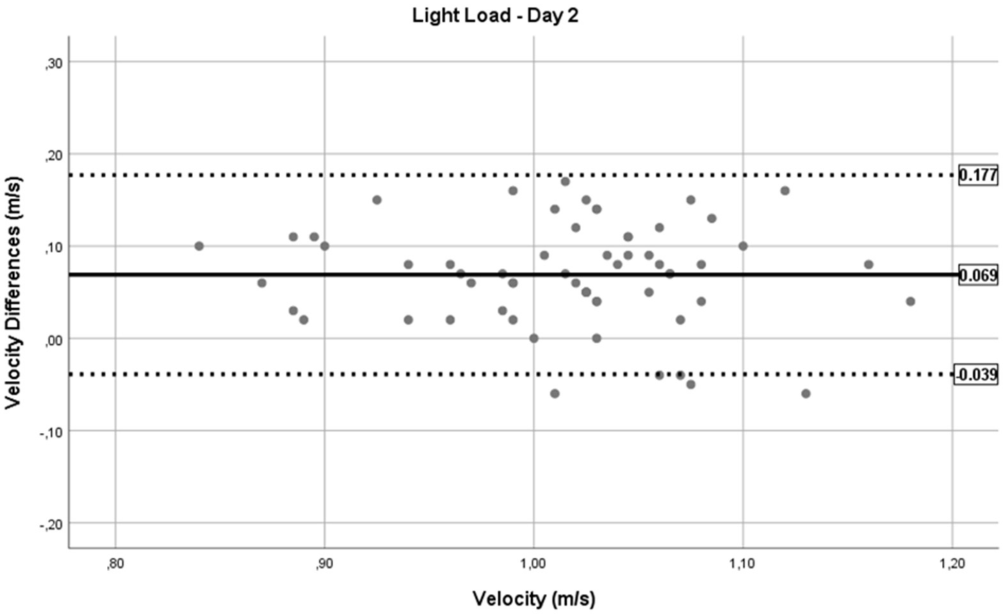

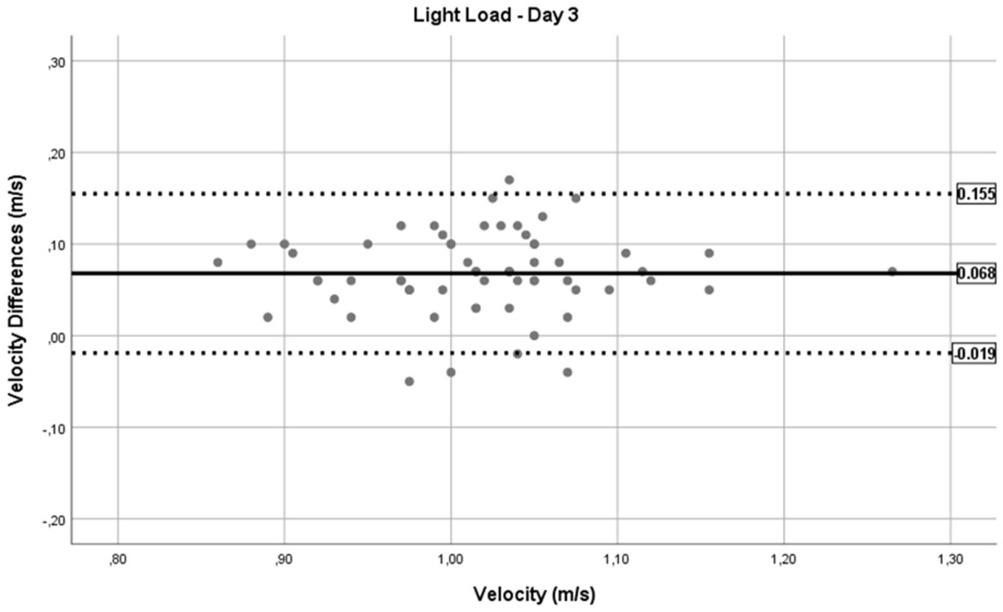
Light Load - Day 3
Velocity Differences (m/s)
,30
,20
,10
,00
-,10
-,20
0.155
0.068
-0.019
,80
,90
1,00
1,10
1,20
1,30
Velocity (m/s)

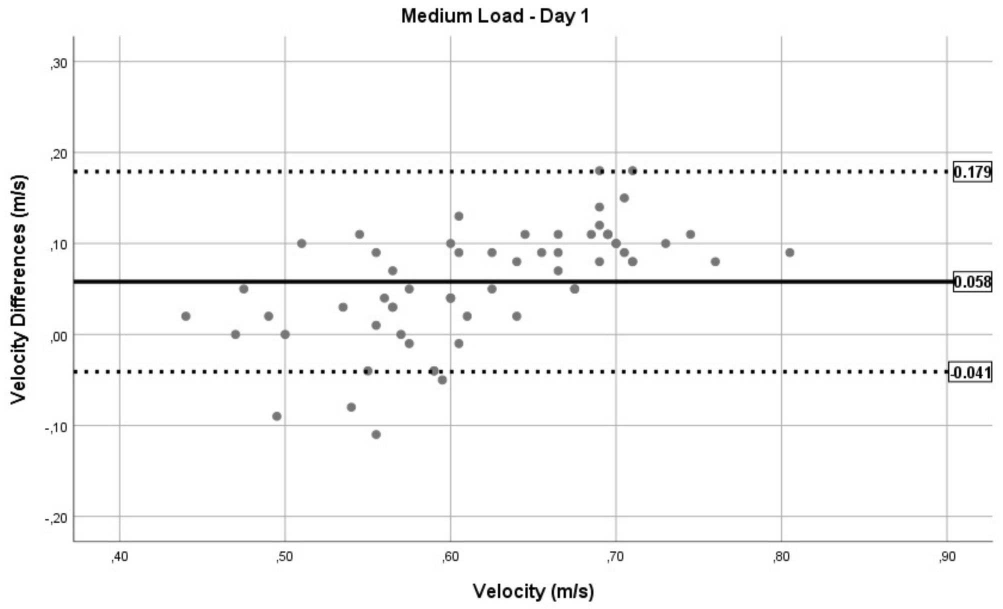
Medium Load - Day 1
Velocity Differences (m/s)
,30
,20
,10
,00
-,10
-,20
0.179
0.058
-0.041
,40
,50
,60
,70
,80
,90
Velocity (m/s)

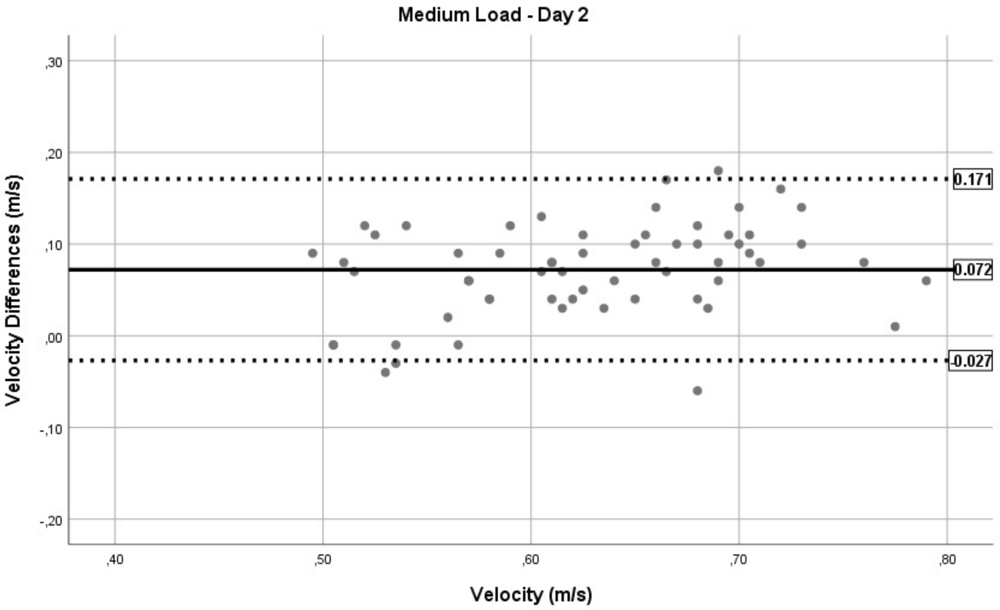
Medium Load - Day 2
Velocity Differences (m/s)
,30
,20
,10
,00
-,10
-,20
0.171
0.072
-0.027
,40
,50
,60
,70
,80
Velocity (m/s)

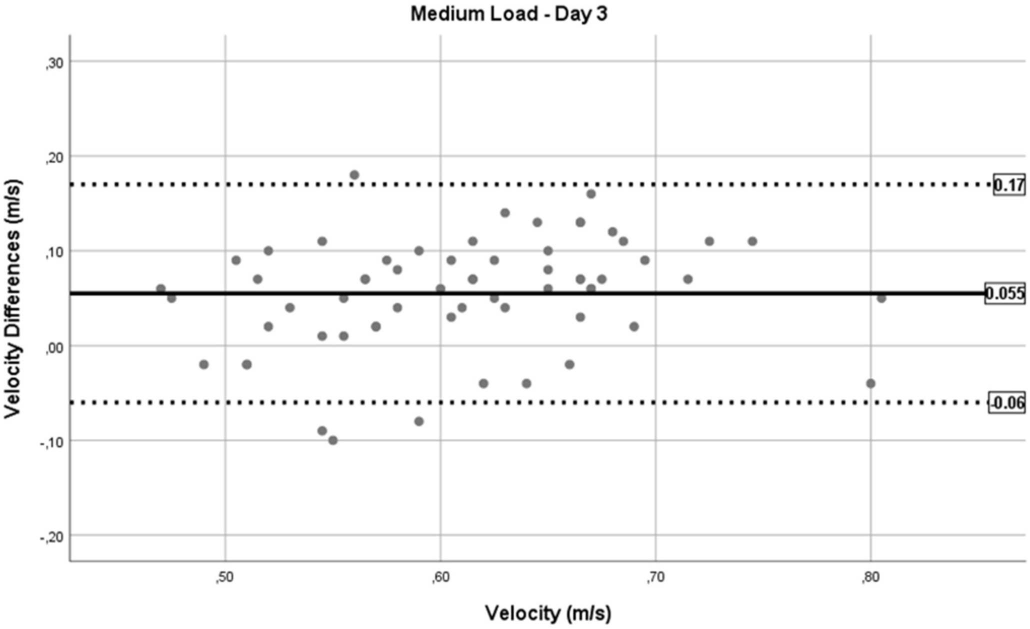
Medium Load - Day 3
Velocity Differences (m/s)
,30
,20
,10
,00
-,10
-,20
0.17
0.055
-0.06
,50
,60
,70
,80
Velocity (m/s)

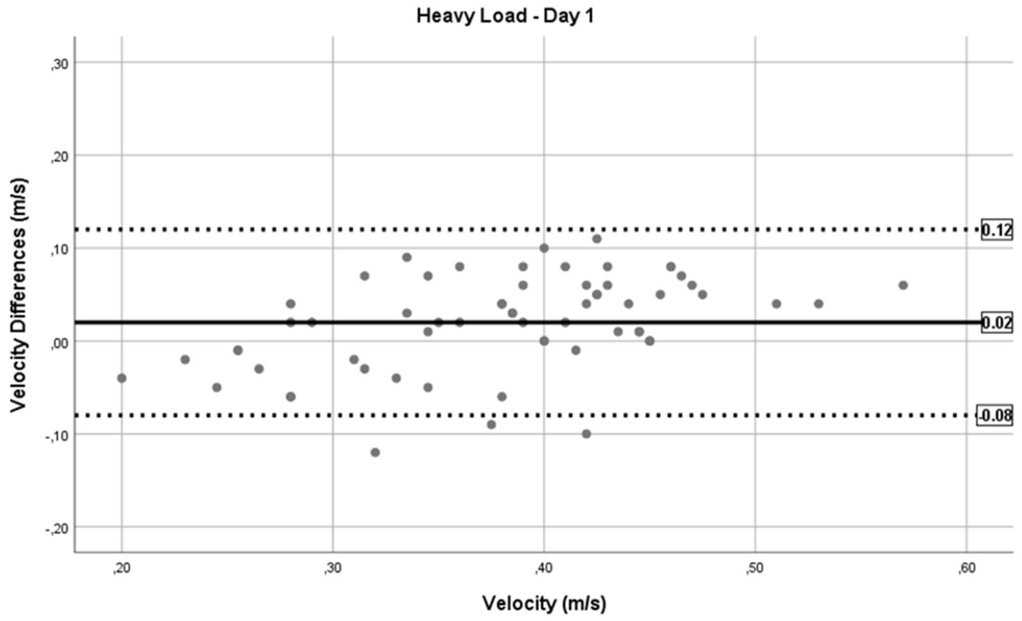
Heavy Load - Day 1
Velocity Differences (m/s)
Velocity (m/s)
,30
,20
,10
,00
-,10
-,20
,20
,30
,40
,50
,60
0.12
0.02
-0.08

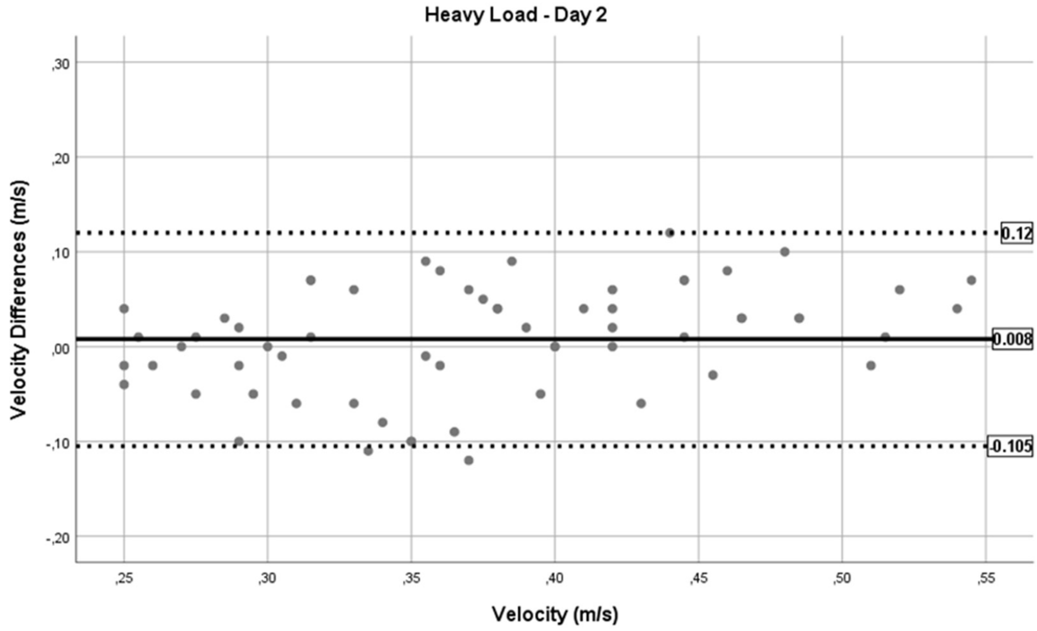
Heavy Load - Day 2
Velocity Differences (m/s)
Velocity (m/s)
,30
,20
,10
,00
-,10
-,20
,25
,30
,35
,40
,45
,50
,55
0.12
0.008
-0.105

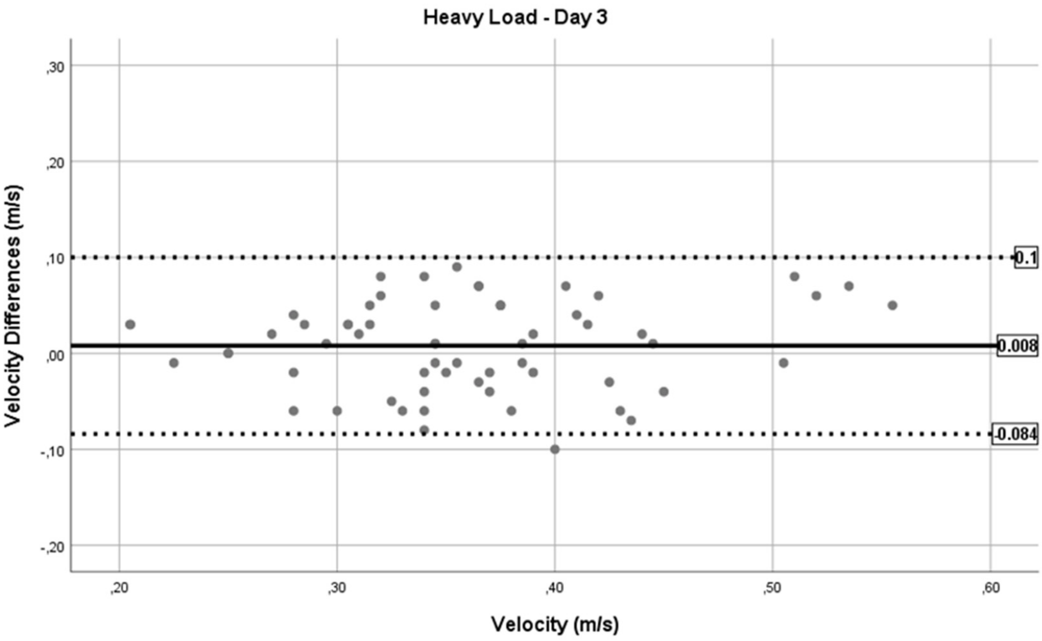

Heavy Load - Day 3
Velocity Differences (m/s)
Velocity (m/s)
,30
,20
,10
,00
-,10
-,20
,20
,30
,40
,50
,60
0.1
0.008
-0.084
